# *De novo* Assembly and Transcriptomic Profiling of the Grazing Response in *Stipa grandis*

**DOI:** 10.1371/journal.pone.0122641

**Published:** 2015-04-13

**Authors:** Dongli Wan, Yongqing Wan, Xiangyang Hou, Weibo Ren, Yong Ding, Rula Sa

**Affiliations:** 1 Institute of Grassland Research, Chinese Academy of Agricultural Sciences, Hohhot, China; 2 College of Life Sciences, Inner Mongolia Agricultural University, Hohhot, China; Chinese Academy of Sciences, CHINA

## Abstract

**Background:**

*Stipa grandis* (Poaceae) is one of the dominant species in a typical steppe of the Inner Mongolian Plateau. However, primarily due to heavy grazing, the grasslands have become seriously degraded, and *S*. *grandis* has developed a special growth-inhibition phenotype against the stressful habitat. Because of the lack of transcriptomic and genomic information, the understanding of the molecular mechanisms underlying the grazing response of *S*. *grandis* has been prohibited.

**Results:**

Using the Illumina HiSeq 2000 platform, two libraries prepared from non-grazing (FS) and overgrazing samples (OS) were sequenced. *De novo* assembly produced 94,674 unigenes, of which 65,047 unigenes had BLAST hits in the National Center for Biotechnology Information (NCBI) non-redundant (nr) database (E-value < 10^-5^). In total, 47,747, 26,156 and 40,842 unigenes were assigned to the Gene Ontology (GO), Clusters of Orthologous Group (COG), and Kyoto Encyclopedia of Genes and Genomes (KEGG) databases, respectively. A total of 13,221 unigenes showed significant differences in expression under the overgrazing condition, with a threshold false discovery rate ≤ 0.001 and an absolute value of log_2_Ratio ≥ 1. These differentially expressed genes (DEGs) were assigned to 43,257 GO terms and were significantly enriched in 32 KEGG pathways (q-value ≤ 0.05). The alterations in the wound-, drought- and defense-related genes indicate that stressors have an additive effect on the growth inhibition of this species.

**Conclusions:**

This first large-scale transcriptome study will provide important information for further gene expression and functional genomics studies, and it facilitated our investigation of the molecular mechanisms of the *S*. *grandis* grazing response and the associated morphological and physiological characteristics.

## Introduction

In China, the grasslands are the dominant landscape, covering 40% of the national land area, of which approximately 78% is located in the northern temperate zone [1]. In the Inner Mongolia Autonomous Region, the steppe grasslands cover 68% of the total land area (up to 791,000 km^2^) [2], but 30–50% of this region is affected by deterioration and desertification [3], and overgrazing is considered to be the major factor contributing to this grassland degradation [2–4].

In the grasslands, grazing is normally linked with plant morphological and physiological responses, such as reduced shoot internodes or altered water utilization rates, to adapt to defoliation disturbance and more stressful habitats [5]. Under grazing conditions, plants are expected to develop a resistance strategy (such as small size, being short-lived and fast-growing or having low palatability) to avoid grazing or a tolerance strategy to re-grow / reproduce after damage; decreased plant height is considered to be a positive response to grazing [6, 7]. The accumulating data have revealed that grazing leads to growth-inhibition in plants; for example, herbivory by the white-tailed deer on Anticosti Island (Quebec) resulted in stunted bonsai-like plants [8]. Additionally, overgrazing by sheep has significantly reduced plant height when compared with non-grazing grasslands [9].

*Stipa grandis* (Poaceae, 2*n* = 44) is a C3 perennial bunch grass and is one of the dominant species in a typical steppe of the Inner Mongolian Plateau [10]. It is a wind-pollinated grass, flowering in mid to late July, with the seeds ripening in late August or early September [10, 11]. The mature plants have dense tussocks that are approximately 30 cm high, with long, thin leaves [11]. The *S*. *grandis* steppe, which represents the major pasture type in Inner Mongolia, primarily spreads from the Xilingole Plateau and the middle-eastern region of the Hulun Buir Plateau [4] and acts as a natural green barrier to protect the vast area from sandstorms [11]. However, as one of the typical dominant steppes in the Xilin River Basin, the *S*. *grandis* steppe has degraded to different degrees, mainly due to overgrazing [4, 12]. As a result, the plants not only showed a smaller size but also reduced plant circumference and sexual reproduction [10].

Because there is no sequencing information for *S*. *grandis* in the public databases, further research on this steppe plant at the molecular level in response to grazing has been limited. Next-generation sequencing (NGS) technologies, such as the Roche / 454, Solexa / Illumina and AB SOLiD platforms [13, 14], have been rapidly developed in recent years, providing more efficient and less costly sequencing than ever before [13]. In the current study, we performed a *de novo* assembly of the leaf transcriptome of *S*. *grandi*s in response to grazing using the Illumina HiSeq 2000 sequencing platform and characterized the transcriptional changes by comparing the transcriptomes of overgrazing and non-grazing plants. This information will facilitate further functional genomics studies in *S*. *grandis* and aid in the understanding of the molecular mechanisms behind the grazing response in plants and the associated morphological changes.

## Materials and Methods

### Ethics statement

Regarding to the field study, no specific permits were required for the *S*. *grandis* species in the described locations in this manuscript, and the field studies did not involve endangered or protected species.

### Plant materials and RNA preparation

The samples used in this experiment were collected from the non-grazing area (referred to as FS) and the overgrazing area (referred to as OS), in a typical dominant steppe in the Baiyinxile Livestock Farm of Xilinhot, Inner Mongolia (116°40'E, 43°33'N), in the middle of August. The non-grazing area has been fenced since 1983 for grazing-free and is considered to be restored, while overgrazing region right next to the fenced area was freely grazed by sheep and seriously degraded [15]. More details about the non-grazing and overgrazing region can be found in previously study [16]. The climate of this area was described previously [2].

For measuring plant height, ten individual plants were randomly chosen and sampled for each region in the morning (over 12 hours after the last grazing happened), and the plants height were statistically analyzed in S1 Fig. For sequencing, three plants were taken from the non-grazing and overgrazing fields each, and fresh leaves of *S*. *grandis* were collected and immediately frozen in liquid nitrogen and then stored at -80°C. 0.1 g leaves of *S*. *grandis* were taken from each plant for total RNA extraction using Trizol regent. The total RNA from each of the three plants was pooled to obtain at least 20 μg of RNA, which was further treated with RNase-free DNase I. The RNA integrity was examined using an Agilent 2100 Bioanalyzer. The poly(A) mRNA was isolated using Oligo(dT) Beads.

### cDNA library construction and sequencing

Following the isolation, the mRNA was fragmented into short fragments using fragmentation buffer. The short fragments were used as templates, and the first-strand cDNA was synthesized using random hexamer primers and reverse transcriptase. Following the second-strand cDNA synthesis, the fragments were end repaired, and poly(A) and sequencing adapters were ligated to the fragments. Following the selection of suitable template fragments, enrichment was performed using PCR amplification to create the final cDNA library. The libraries were sequenced using an Illumina HiSeq 2000.

### Data filtering and *de novo* assembly

The raw reads were filtered by removing the reads containing adaptor sequences, “N” (unknown nucleotides) percentages that were greater than 5%, and low-quality reads (the rate of reads which quality value ≤ 10 is more than 20%) with software “filter_fq”. Then, the transcriptome *de novo* assembly was performed using the short-read assembly program Trinity [17]. Trinity performs analysis in three steps using different software modules: Inchworm, Chrysalis, and Butterfly. Briefly, Inchworm assembles the cleaning reads into the linear transcript contigs (unique sequences of transcripts) using greedy K-mer extension (k = 25). Then Chrysalis clusters minimally overlapping Inchworm contigs into clusters and builds complete de Bruijn graphs for each cluster, representing the full transcriptional complexity for a given gene. In the last step, Butterfly processed the individual graphs in parallel, compacted the graphs and report full-length transcripts for alternatively spliced isoforms and paralogous transcripts. The sequences generated from Trinity were defined as unigenes. The assembled unigenes from FS and OS were used for further sequence splicing and redundancy removing with clustering software TGICL [18] to get non-redundant unigenes as long as possible. After gene family clustering, the final obtained unigenes were divided into either clusters (shared more than 70% similarity) or singletons. Finally, a Blastx [19] alignment (E-value < 10^-5^) was performed between the unigenes and various protein databases, such as the non-redundant protein (nr) database (http://www.ncbi.nlm.nih.gov), the Swiss-Prot protein database (http://www.expasy.ch/sprot), the Kyoto Encyclopedia of Genes and Genomes (KEGG) pathway database (http://www.genome.jp/kegg) and the Cluster of Orthologous Groups (COG) database (http://www.ncbi.nlm.nih.gov/COG). The best aligning results were used to decide the unigene’s sequence orientation. A priority order of nr, Swiss-Prot, KEGG and COG was used when the outcome from the different databases conflicted. If a unigene did not align to any of the above databases, ESTScan [20] was used to predict its sequence orientation.

### Unigene annotation and classification

For the annotation, the unigenes were first aligned using Blastx against the protein databases, including nr, Swiss-Prot, KEGG and COG (E-value < 10^-5^). The protein with the highest sequence similarity was retrieved using the given unigenes and was annotated to each unigene. The GO (gene ontology; http://www.geneontology.org) annotations for the unigenes were performed using the Blast2GO [21] program, based on the best Blastx hits from the nr database (E-value < 10^-5^), and the GO functional classification for All-unigenes was performed using WEGO [22]. The COG database was also used for possible functional prediction and classification of the unigenes. The pathway assignments were generated using Blastall software against the KEGG database.

### Estimation of the expression levels and the differential expression analyses

To calculate the unigene expression levels, the FPKM method (Fragments Per kb per Million fragments) was employed, as previously described [23]. The fold changes of the transcripts were calculated using the log_2_ formula of OS_ FPKM / FS_FPKM, and 0.01 was used (instead of 0) for the fold change calculation once the value of either OS_ FPKM or FS_FPKM was zero. The differentially expressed genes (DEGs) between the two samples were analyzed with a rigorous algorithm based on Audic’s [24] method. The false discovery rate (FDR) method [25] was used to correct for the P-value in the multiple hypothesis testing. An FDR ≤ 0.001 and an absolute value of log_2_Ratio ≥ 1 were used as the thresholds to judge the significance of the differential gene expression. For the functional and pathway enrichment analysis, the DEGs were then mapped into GO terms (P-value ≤ 0.05) and the KEGG database (q-value ≤ 0.05).

### Quantitative real-time PCR analysis (qPCR)

The qPCR was performed using a Roche LightCycler 480 Real-Time PCR system, as previously described [26]. *Actin* was used as the internal control gene, and the 2^-ΔΔCT^ method [27] was used to evaluate the relative quantities of each amplified product in the samples. For each qPCR analysis, three technical replicates were performed. The primers used for the qPCR were provided in S1 Table.

### Data deposition

The clean raw reads data were deposited in NCBI Sequence Read Archive (SRA, http://www.ncbi.nlm.nih.gov/Traces/sra) under the accession number SRP051667.

## Results

### Illumina sequencing and reads assembly

To investigate the transcriptomic response of *S*. *grandis* to overgrazing, the leaves of *S*. *grandis* from both FS and OS areas were sampled. When compared with the non-grazing plants, the *S*. *grandis* under the overgrazing conditions were significantly reduced in height (S1 Fig). In total, 77,859,274 raw reads for FS and 75,960,480 raw reads for OS were generated using the Illumina HiSeq 2000 sequencing platform. After filtering out the dirty raw reads, 65,954,618 and 64,545,856 clean reads (with an average length of 90 bp) were obtained from the FS and OS samples (Table 1), respectively.

**Table 1:** A summary of the transcriptome sequencing and assembly results in the FS and OS populations of *S*. *grandis*.

	Sample	Total Number	Total Length (nt)	Mean Length (nt)	N50 (bp)
**Raw reads**	FS	77,859,274			
	OS	75,960,480			
**Clean reads**	FS	65,954,618			
	OS	64,545,856			
**Contig**	FS	193,974	53,395,584	275	375
	OS	184,321	50,894,860	276	368
**Unigene**	FS	94,905	61,536,711	648	1130
	OS	93,816	59,020,466	629	1103
	All-unigenes	94,674	73,795,643	779	1271

Mean length: Mean length of the assembled sequences. N50: The length of the contig or unigene corresponding to the sequence, which is added to 50% of the total assembled bases when the assembled sequences are sorted from short to long.

**Fig 1:**
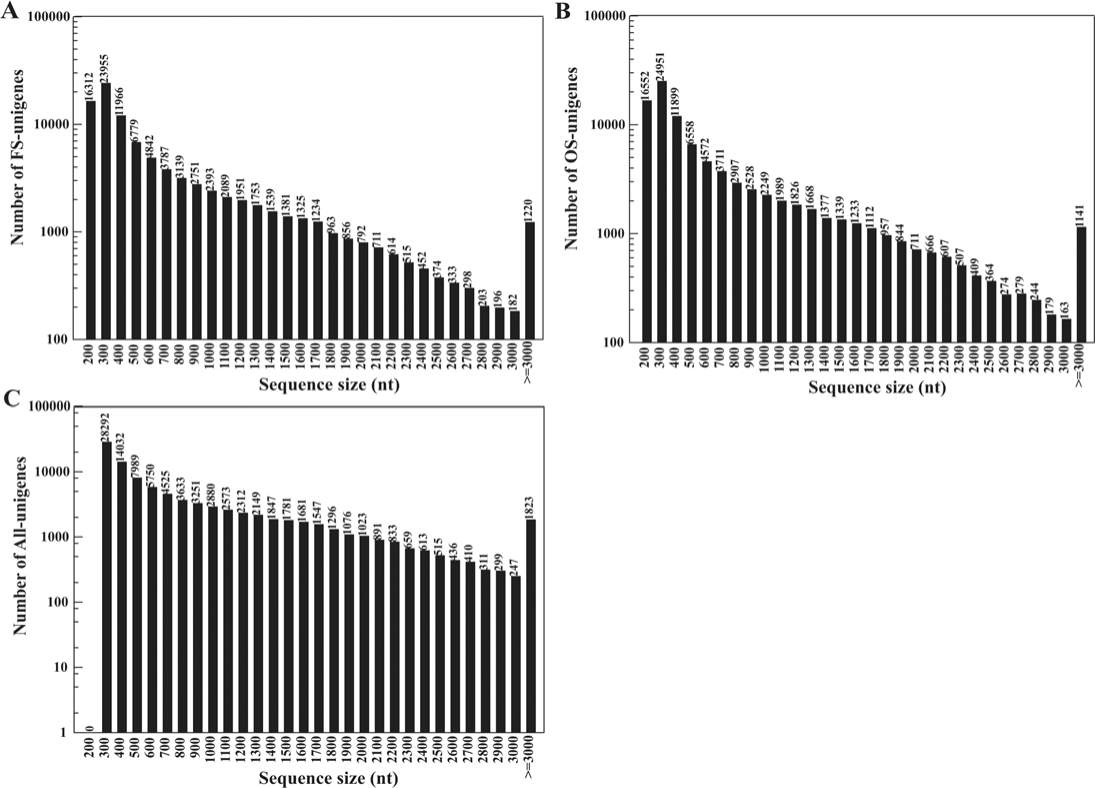
Length distributions of the FS-, OS- and All-unigenes. Length distributions of (A) the FS-unigenes, (B) the OS-unigenes, and (C) All-unigenes. The y-axis indicates the number of unigenes, and the x-axis indicates the sequence sizes of the unigenes.

The Trinity [17] method was adopted to assemble all of the high-quality clean reads into contigs and unigenes. As a result, 193,974 FS contigs and 184,321 OS contigs were generated (Table 1). The majority of the contigs from both samples had similar length distributions in the range of 100–300 bp (S2 Fig). The above contigs were further assembled into 94,905 FS-unigenes and 93,816 OS-unigenes. Notably, all of the unigenes were gap-free sequences. Ultimately, 94,674 unigenes, with a mean length of 779 bp and an N50 of 1271 bp, were obtained by combining the FS and OS unigenes (Table 1), including 44,421 clusters and 50,253 singletons. As shown in Fig 1, the FS and OS unigenes shared similar length distributions; the highest sequence range proportion was 100–500 bp, with 62.18% (59,012 / 94,905) for FS and 63.91% (59,960 / 93,816) for OS. However, All-unigenes dramatically increased in length when the 100-200-bp regions were removed and only the unigenes with long sequences were included; the length distribution percentages increased to 74.31% (70,352 / 94,674) in the 200-1000-bp range. These results suggest that our transcriptome sequencing data were assembled effectively.

### Functional annotation of the assembled unigenes

**Fig 2:**
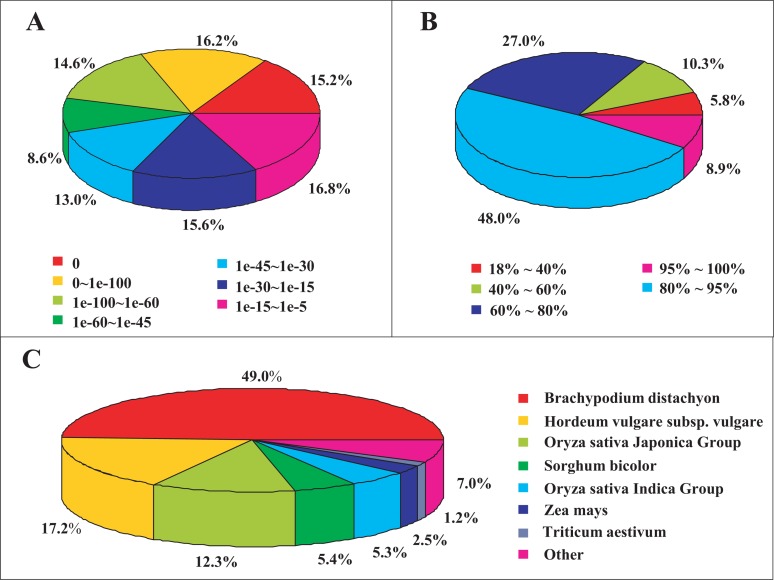
Unigene homology searches against the nr database. (A) The proportional frequency of the E-value distribution. (B) The proportional frequency of the sequence similarity distribution. (C) The proportional species distribution of *S*. *grandis* among other plant species.

For the functional annotation, the unigene sequences were first blasted against the NCBI nr database using Blastx (E-value < 10^-5^). Of the 94,674 unigenes, 65,047 (68.71%) were annotated (Table 2), whereas 29,627 unigenes (31.29%) were not matched to any known proteins in the nr database. The E-value frequency distribution analysis revealed that 46% of the sequences shared strong homologies, with E-values ≤ 1.0E-60, while the remaining 54% fell into the range of 1.0E-60–1.0E-5 (Fig 2A). Furthermore, we also observed that 56.9% of the sequences had a similar distribution range between 80% and 100%, but only 5.8% had similarity values less than 40% (Fig 2B). Based on the homologous species identified among the annotated unigenes, 49.0% of the unigene sequences matched to *Brachypodium distachyon*, followed by *Hordeum vulgare subsp*. *vulgare* (17.2%) and *Oryza sativa Japonica* (12.3%) (Fig 2C). In addition to the nr annotation, 42,233 unigenes (44.61%) were aligned to known proteins in the Swiss-Prot database (E-value < 10^-5^) (Table 2).

**Table 2:** A summary of the functional annotations of the assembled unigenes.

Public protein database	Number of unigene hits	Percentage (%)^a^
**NR**	65,047	68.71%
**Swiss-Prot**	42,233	44.61%
**GO**	47,747	50.43%
**COG**	26,156	27.63%
**KEGG**	40,842	43.14%

^a^ Represents the proportion of the 94,674 assembled unigenes.

**Fig 3:**
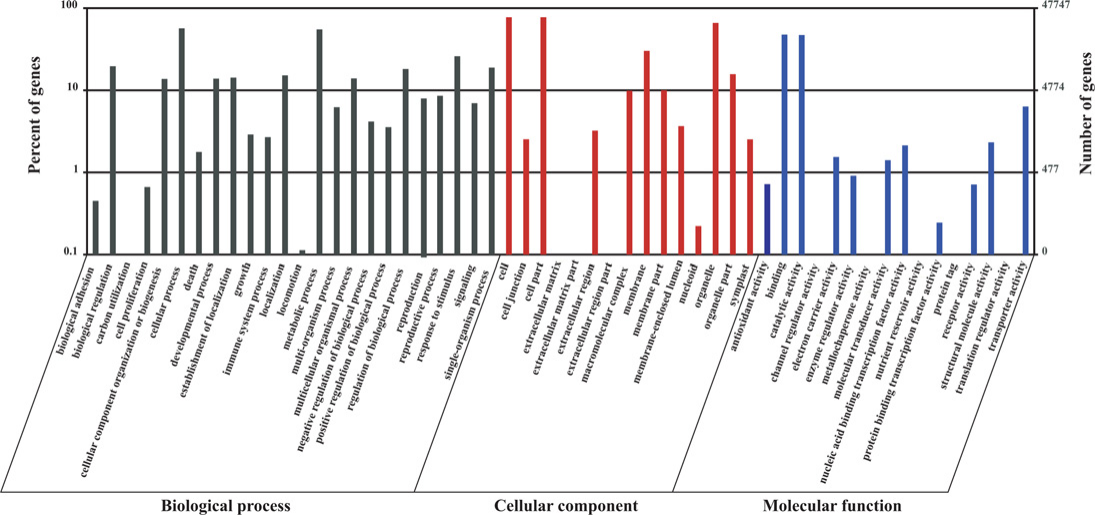
Histogram of the GO (gene ontology) classification of the *S*. *grandis* unigenes. All 47,747 unigenes were grouped into three ontologies: molecular function, cellular component and biological process. The y-axis indicates the percentage (left) and number (right) of genes in each term.

To further functionally categorize the *S*. *grandis* unigenes, the genes were matched to the international standardized gene functional classification system (GO). A total of 47,747 unigenes (Table 2) were assigned to one or more GO terms and categorized into 55 functional groups (Fig 3), which belong to three main GO ontologies: molecular function, cellular component and biological process. The results showed that a high percentage of genes were assigned to ‘‘cell”, ‘‘cell part”, ‘‘organelle”, ‘‘membrane”, ‘‘catalytic activity”, ‘‘binding”, ‘‘metabolic process”, ‘‘cellular process” and ‘‘response to stimulus”. However, a few genes were clustered as ‘‘channel regulator activity”, ‘‘metallochaperone activity”, ‘‘nutrient reservoir activity”, ‘‘protein tag”, ‘‘translation regulator activity”, ‘‘extracellular matrix” or ‘‘extracellular matrix part”, ‘‘extracellular region part”, “carbon utilization” and ‘‘locomotion”.

**Fig 4:**
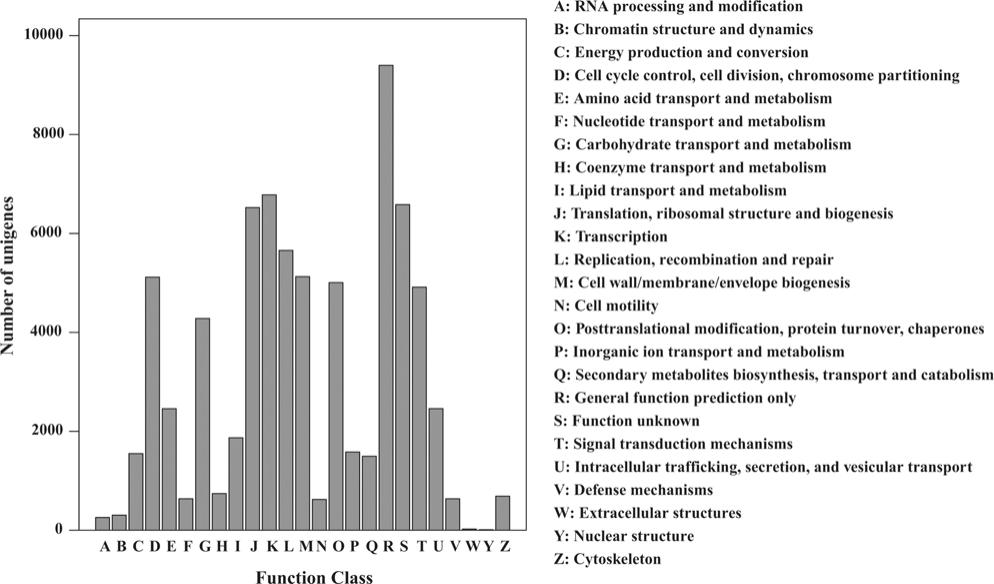
Histogram of the COG (clusters of orthologous groups) functional classification of all of the *S*. *grandis* unigenes. Out of the 94,674 *de novo* assembled unigenes, 26,156 were annotated and grouped into 25 categories.

In an attempt to further evaluate the integrality of our transcriptome library and the effectiveness of the annotation process, COG was used to classify the unigenes. In total, 26,156 unigenes (Table 2) were divided into 25 COG categories (Fig 4), of which the largest group was the cluster ‘‘general function prediction” (9399), followed by ‘‘transcription” (6781), ‘‘function unknown” (6583), ‘‘translation, ribosomal structure and biogenesis” (6523) and ‘‘replication, recombination and repair” (5657). ‘‘Nuclear structure” (9), ‘‘extracellular structures” (23) and ‘‘RNA processing and modification” (257) represented the smallest groups. To further investigate their biological functions, the unigenes were mapped to reference canonical pathways in KEGG. As a result, 40,842 unigenes (Table 2) were KEGG annotated and assigned to 128 pathways.

### Differential expression and pathway analyses in the FS and OS populations

**Fig 5:**
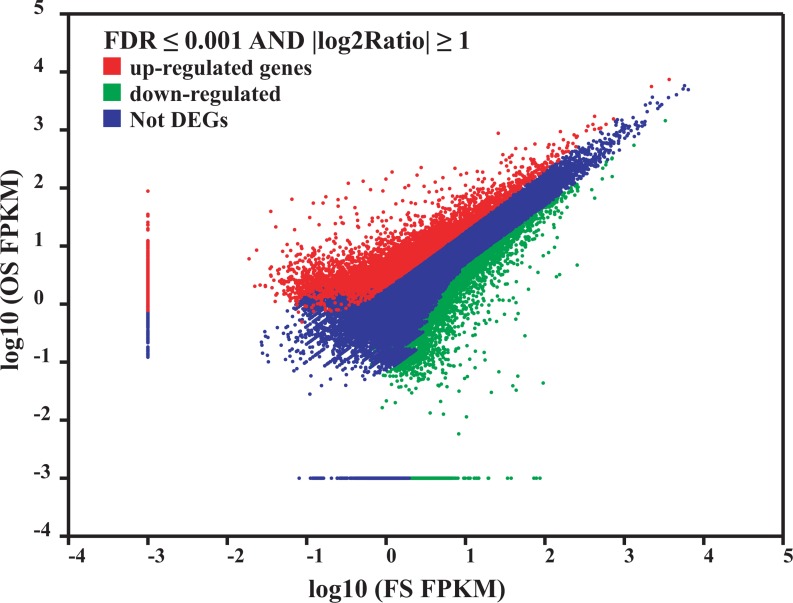
Identification of the DEGs (differentially expressed genes) between FS and OS. The DEGs were determined using a threshold of FDR ≤ 0.001 and an absolute value of log_2_Ratio ≥ 1. The red, green and blue spots represent the upregulated, the downregulated DEGs and the genes without obvious changes in response to grazing, respectively.

To reveal the differential expression profiles between FS and OS, the potential DEGs were analyzed. The FPKM method [23] was used to calculate the expression levels. When a threshold of FDR ≤ 0.001 and an absolute value of log_2_Ratio ≥ 1 were used, a total of 13,221 unigenes showed significant differences in response to grazing, of which 6283 unigenes were upregulated and 6938 unigenes were downregulated (Fig 5).

**Fig 6:**
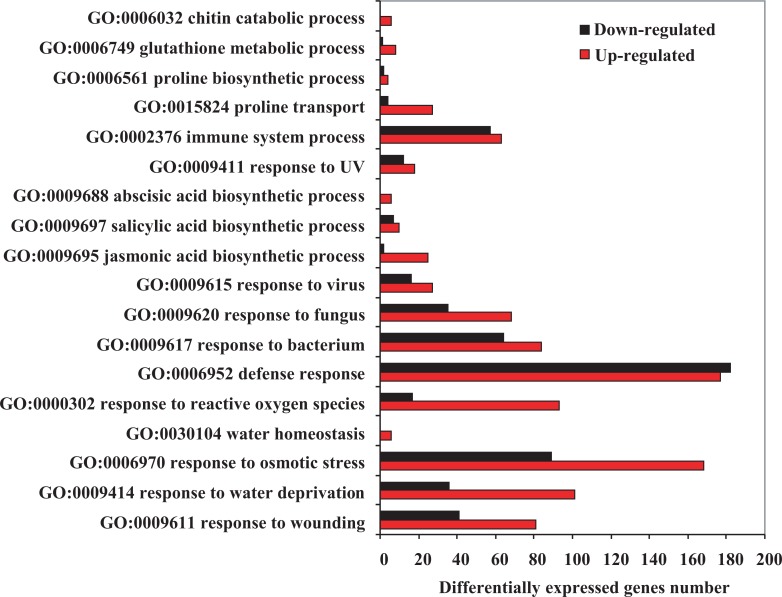
The GO term assignments for the DEGs related to the stress response under the overgrazing and non-grazing conditions. The red bars represent the upregulated DEGs, the black bars represent the downregulated DEGs.

Base on the nr annotation, a total of 6526 DEGs were assigned 43,257 GO terms and classified into 51 functional categories, which belongs to three main ontologies: biological process (17,746), cellular component (18,307) and molecular function (7204) (S2 Table). By analyzing the “biological process” categories, 18 subcategories, such as “response to wounding” (GO:0009611), “response to water deprivation” (GO:0009414), “response to reactive oxygen species” (GO:0000302), “defense response” (GO:0006952), “proline biosynthetic process” (GO:0006561) and “chitin catabolic process” (GO:0006032), which is related to resistant responses to abiotic and biotic stressors, were observed (Fig 6).

To further explore the biological functions of the DEGs, a pathway enrichment analysis was performed. A total of 8747 DEGs were enriched in 32 metabolic pathways (q-value ≤ 0.05), and the top enriched metabolic pathways included “plant-pathogen interaction” (675 DEGs), “RNA transport” (951 DEGs), and “mRNA surveillance pathway” (830 DEGs) (S2 Table).

### Validation of the transcriptome results via qPCR analysis

**Fig 7:**
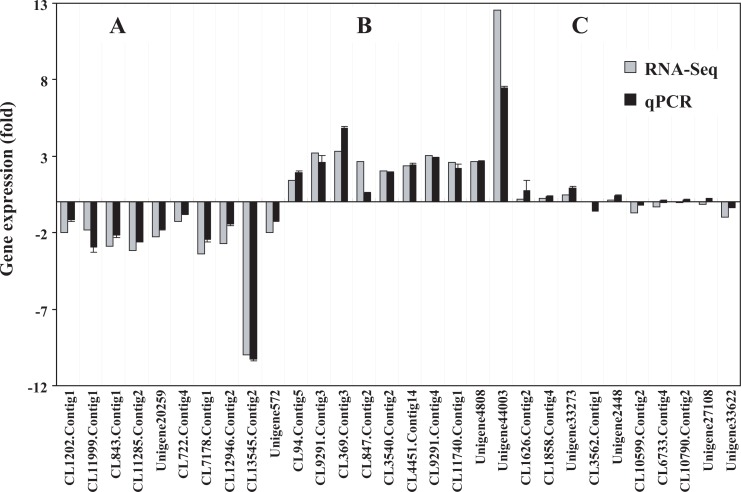
qPCR validation of the gene expression patterns. A total of 30 genes, including (A) 10 downregulated, (B) 10 upregulated, and (C) 10 unigenes with no difference in expression and with an absolute value of log_2_Ratio < 1, were selected, and the expression levels were confirmed using qPCR. The gray bars represent the changes in transcript abundance determined by the FPKM method. The black bars represent the relative expression levels estimated using qPCR and the 2^-ΔΔCT^ method. The error bars indicate the standard deviation (n = 3).

To validate the expression levels in the transcriptome, 30 genes exhibiting different expression patterns (10 upregulated, 10 downregulated, and 10 unigenes with no obvious changes in transcript abundance, with a |log_2_Ratio| < 1 between FS and OS) were randomly selected and examined using qPCR. As a result, the relative expression patterns of the 30 unigenes tested via qPCR correspond well with the transcriptome data that was determined using the FPKM method, exhibiting clear up- or down-regulation or no change in response to grazing (Fig 7), suggesting that the transcriptomic profiling data were reliable. However, some quantitative differences in the relative expression levels were observed, which may be due to the differential sensitivities of the two techniques.

### Genes related to stress and defense

Grazing is a process containing multiple stressors, involving both biotic and abiotic stressors and including trampling / wounding, water stress and pathogen infection.

Because JAs (jasmonates) play a key role in the wound response [28], 52 DEGs involved in the JA biosynthesis and signaling pathway, along with those regulated by wounds, were identified, of which 38 genes were upregulated and the other 14 were downregulated in response to grazing (Table 3 and S3 Table). The genes involved in JA biosynthesis included *AOS* (*allene oxide synthase*, also known as *CYP74A*) [29], *LOX* (*lipoxygenase*) [30], *OPR* (*12-oxophytodienoate reductase*) [31], *FAD* (*Fatty acid desaturase*) [32] and *PLD* (*phospholipase D*) [33]. Other genes that are relevant to the JA signaling pathway included *JAZ* (jasmonate ZIM domain) */ TIFY* and *COI1* (*coronatine insensitive 1*) [34, 35].

**Table 3:** The DEGs related to abiotic stress.

Genes	Gene ID	FS FPKM	OS FPKM	Fold change	FDR	Genes	Gene ID	FS FPKM	OS FPKM	Fold change	FDR
**AOS**	CL12787.Contig1	2.78	18.15	2.71	4.49E-48	**JAZ / TIFY**	CL513.Contig5	8.37	39.47	2.24	1.86E-122
	CL9995.Contig1	0.81	6.61	3.02	5.42E-44		CL513.Contig3	9.79	42.01	2.10	2.25E-116
	Unigene7257	32.08	8.54	-1.91	4.58E-35		CL513.Contig2	9.73	33.12	1.77	9.82E-84
	CL9995.Contig2	0.83	5.89	2.83	7.11E-35		CL513.Contig1	6.11	21.36	1.80	1.10E-56
**LOX**	CL7512.Contig1	2.51	32.91	3.71	0		CL513.Contig4	4.23	10.46	1.31	4.68E-23
	CL789.Contig2	4.33	87.26	4.33	6.07E-142		CL15037.Contig1	10.40	22.98	1.14	3.13E-40
	CL9755.Contig1	2.34	53.58	4.52	2.49E-85	**DREB**	CL9452.Contig2	30.09	10.81	-1.48	1.24E-73
	CL7512.Contig2	4.99	11.04	1.14	6.90E-11		CL9452.Contig1	28.35	11.09	-1.35	1.98E-62
	Unigene11705	2.86	6.82	1.25	7.89E-07		CL1929.Contig6	8.16	20.68	1.34	9.79E-57
**FAD**	CL40.Contig4	245.67	515.41	1.07	0		CL1929.Contig8	5.72	15.96	1.48	1.16E-50
	CL40.Contig1	8.69	21.69	1.32	5.58E-24		CL1929.Contig5	5.13	12.43	1.28	9.14E-41
	Unigene22017	22.84	46.65	1.03	6.55E-22		CL1929.Contig9	4.45	10.82	1.28	9.95E-35
	CL491.Contig1	0.07	2.67	5.28	4.38E-10		CL13869.Contig1	8.48	17.39	1.04	2.97E-11
	Unigene40701	0.00	2.22	11.12	8.11E-08	**NCED**	CL8856.Contig4	19.29	39.22	1.02	2.98E-105
	CL5434.Contig3	3.13	7.51	1.26	2.00E-07		CL8856.Contig1	11.30	30.62	1.44	4.62E-31
	Unigene12113	7.90	3.22	-1.30	5.07E-05		CL12565.Contig1	1.88	6.31	1.74	4.06E-08
**OPR**	CL5872.Contig1	192.88	537.10	1.48	0		CL1476.Contig1	3.54	7.13	1.01	6.82E-07
	CL5872.Contig4	166.32	430.50	1.37	0	**Dehydrin**	CL4123.Contig1	145.71	362.95	1.32	0
	CL5872.Contig8	140.87	377.14	1.42	0		CL4123.Contig3	47.86	192.08	2.00	0
	CL5170.Contig2	4.40	1.60	-1.46	1.06E-05		CL4123.Contig4	43.21	164.64	1.93	0
	CL5872.Contig7	0.79	3.41	2.11	5.39E-05		CL5735.Contig3	57.07	139.37	1.29	1.62E-212
**PLD**	CL15321.Contig2	3.75	8.26	1.14	4.37E-14		Unigene15994	20.98	82.84	1.98	9.83E-204
	CL112.Contig3	5.12	12.61	1.30	5.36E-93		CL5735.Contig2	42.40	102.93	1.28	2.24E-126
	CL3882.Contig2	4.86	1.77	-1.46	8.21E-05		Unigene33265	3.82	0.45	-3.08	5.03E-18
	Unigene32822	2.50	0.80	-1.64	1.05E-05	**LEA**	Unigene15689	1.62	60.39	5.22	0
	Unigene7167	2.36	0.70	-1.76	1.39E-04		Unigene24764	37.15	277.73	2.90	0
	CL3882.Contig1	5.07	1.98	-1.35	5.42E-04		Unigene9383	17.29	51.87	1.59	8.80E-90
**COI1**	CL13823.Contig1	0.85	3.28	1.95	1.60E-14		CL3750.Contig1	15.01	37.86	1.33	8.90E-52
	CL6692.Contig6	1.22	3.21	1.39	2.59E-11		CL6420.Contig4	2.71	12.32	2.18	6.91E-19
	CL6692.Contig2	1.00	2.32	1.21	7.71E-07		Unigene19508	4.86	2.36	-1.04	2.40E-07
	Unigene18713	13.46	5.73	-1.23	4.60E-20		CL6420.Contig2	2.36	4.85	1.04	6.03E-05
	Unigene17090	13.98	6.69	-1.06	6.67E-11		Unigene8570	0.62	3.20	2.36	1.44E-04

The prefix “CL” represents clusters, and “unigene” represents singletons. FPKM indicates the FPKM values of the unigenes in FS or OS. “Fold change” is equal to log_2_ (OS-FPKM / FS-FPKM). “+” indicates upregulated transcription, and “-” represents downregulated transcription.

Based on previously published data [36–39], we searched for water-deprivation response genes in our grazing-regulated DEGs, such as the *LEA* (*late embryogenesis abundant*), *DREB* (*dehydration-responsive element-binding*), *NCED* (*9-cis-epoxycarotenoid dioxygenase*) and *dehydrin* genes. We found that the majority of the transcripts (76.9%, 30 / 39) of these genes were increased by grazing (Table 3 and S3 Table). Together, these results suggest that grazing induces wound- and water-stress response of *S*. *grandis*.

Overall, we identified a total of 115 defense-response related DEGs among the 675 DEGs in the plant-pathogen interaction pathway (Table 4 and S3 Table), including the pathogenesis-related gene *PR1* (Unigene6840), which is considered to be one of the SAR (systemic acquired resistance) marker genes [40], and one *PRB1-2*-like gene (Unigene2461), which had accumulated in the OS sample, indicating that disease resistance was activated. Six leucine-rich repeat receptor kinase (LRR-RK) transcripts, encoding the *FLS2* (*flagellin sensitive 2*) genes [41], and 11 *RPM1* (*resistance to Pseudomonas maculicula protein 1*) [42] were increased by overgrazing. In contrast, 4 homologs of *RIN4* (encoding an RPM1-interacting protein) [42], showed attenuated expression. Additionally, the transcripts of the *SGT1* (suppressor of G-two allele of *Skp1*) [43] and *Hsp90* (*heat shock protein* 90) genes were significantly enhanced by grazing. Notably, a set of *WRKY* genes, including *WRKY-1*, *-7*, *-11*, *-55*, and *-70*, also exhibited altered expression due to overgrazing. These results indicate that the immune response of *S*. *grandis* is enhanced and that disease resistance is activated by overgrazing.

**Table 4:** The DEGs related to defense.

Genes	Gene ID	FS FPKM	OS FPKM	Fold change	FDR	Genes	Gene ID	FS FPKM	OS FPKM	Fold change	FDR
**PR1**	Unigene6840	7.12	18.88	1.41	2.84E-28	**SGT1**	CL3879.Contig2	18.93	47.68	1.33	3.11E-195
	Unigene2461	3.16	7.18	1.18	2.46E-04		CL3879.Contig1	33.79	71.54	1.08	4.84E-150
**FLS2**	Unigene43887	0.15	2.50	4.04	5.06E-04	**WRKY6**	CL178.Contig2	16.26	6.01	-1.44	2.94E-63
	Unigene43888	0.45	3.06	2.76	8.10E-09		CL178.Contig1	17.39	6.67	-1.38	1.04E-63
	Unigene12048	0.63	3.83	2.60	6.61E-11	**WRKY3**	CL6449.Contig1	7.00	2.75	-1.35	1.61E-20
	Unigene2737	0.66	3.76	2.51	1.40E-24		CL6449.Contig3	5.78	2.13	-1.44	1.31E-20
	Unigene11377	0.53	2.41	2.18	2.35E-04		CL6449.Contig4	4.14	1.68	-1.30	1.07E-13
	CL3221.Contig2	1.22	3.95	1.69	2.18E-09		CL6449.Contig2	12.46	4.99	-1.32	3.03E-37
**RPM1**	CL9260.Contig3	0.07	3.64	5.71	1.73E-27		CL6449.Contig5	3.80	1.63	-1.22	2.83E-10
	CL13422.Contig1	0.00	2.92	11.51	2.43E-14	**WRKY20**	CL511.Contig1	34.97	14.03	-1.32	2.40E-130
	CL11573.Contig2	0.98	3.06	1.65	2.98E-10		CL511.Contig9	27.55	11.02	-1.32	1.07E-108
	Unigene40051	0.00	2.99	11.55	7.08E-10		CL511.Contig2	35.84	15.82	-1.18	3.09E-113
	Unigene17462	1.93	4.93	1.36	1.79E-09		CL511.Contig8	32.29	13.68	-1.24	2.53E-115
	Unigene38815	0.09	2.58	4.89	1.54E-07		CL511.Contig5	26.03	11.33	-1.20	1.54E-89
	CL15353.Contig1	0.00	1.90	10.89	8.78E-06		CL511.Contig6	27.04	11.47	-1.24	5.94E-97
	CL1475.Contig1	0.72	2.40	1.74	3.07E-05	**WRKY71**	CL2147.Contig3	7.14	3.18	-1.17	2.33E-11
	CL467.Contig1	1.61	3.56	1.15	3.60E-05		CL2147.Contig1	22.34	5.57	-2.00	1.75E-72
	Unigene39466	1.10	3.34	1.60	4.78E-05	**WRKY21**	Unigene20256	20.10	7.28	-1.47	6.58E-98
	Unigene42608	0.00	1.60	10.64	6.33E-05	**WRKY11**	CL4223.Contig1	28.86	11.56	-1.32	4.59E-13
	CL7239.Contig3	3.64	0.93	-1.96	3.19E-44	**WRKY58**	CL4473.Contig1	13.82	5.23	-1.40	1.20E-47
	Unigene17990	13.76	0.86	-3.99	8.50E-42		CL4473.Contig3	29.24	9.84	-1.57	6.80E-116
	CL7239.Contig4	3.52	1.03	-1.77	1.51E-37	**WRKY7**	CL168.Contig3	41.47	15.13	-1.45	1.86E-59
	CL15522.Contig1	7.39	1.89	-1.97	2.12E-36		CL168.Contig4	21.19	7.30	-1.54	6.12E-44
	CL3622.Contig6	5.28	2.28	-1.21	5.23E-32	**WRKY1**	CL14068.Contig1	55.85	11.28	-2.31	8.47E-132
	CL4636.Contig4	4.87	2.39	-1.03	3.07E-24		CL14068.Contig2	28.14	10.42	-1.43	6.99E-39
	CL11476.Contig2	3.42	0.19	-4.13	6.12E-16		Unigene18532	6.58	2.33	-1.50	1.04E-11
	Unigene30488	2.44	0.00	-11.25	2.99E-13		CL5938.Contig1	9.58	3.95	-1.28	4.22E-28
	CL11573.Contig1	3.17	0.46	-2.80	2.92E-09		CL1657.Contig4	212.46	101.23	-1.07	0
	Unigene7686	5.43	2.48	-1.13	3.48E-07	**WRKY55**	CL2736.Contig5	1.12	5.94	2.41	5.91E-31
**RIN4**	CL1992.Contig2	0.75	1.72	1.21	5.88E-04		CL2736.Contig6	0.73	3.43	2.24	4.89E-18
	CL10916.Contig3	51.66	19.81	-1.38	6.30E-122		CL2736.Contig3	2.00	4.43	1.14	1.02E-07
	CL10916.Contig4	36.82	15.49	-1.25	2.63E-127		CL2736.Contig9	0.74	5.88	2.99	1.78E-42
	CL10916.Contig5	32.11	14.17	-1.18	3.41E-54		CL2736.Contig1	1.31	9.04	2.79	7.72E-56
	CL10916.Contig6	26.19	12.78	-1.04	8.63E-64		CL2736.Contig4	1.48	6.52	2.14	6.18E-28
**HSP90**	CL898.Contig1	0.25	48.67	7.61	0		Unigene17732	0.50	2.79	2.47	6.72E-17
	CL898.Contig4	0.14	23.27	7.34	6.49E-92		CL11908.Contig1	12.45	6.17	-1.01	2.85E-18
	CL898.Contig2	0.12	8.58	6.10	5.98E-37	**WRKY33**	Unigene4825	44.30	7.94	-2.48	2.91E-73
	CL15329.Contig2	0.09	3.80	5.36	1.17E-10		Unigene4405	81.66	24.90	-1.71	3.21E-73
	Unigene45696	0.65	14.04	4.43	8.99E-65		CL5906.Contig2	63.08	12.53	-2.33	1.29E-182
	CL15329.Contig1	0.73	6.78	3.22	8.21E-27		CL5906.Contig1	78.48	23.50	-1.74	2.23E-157
	CL898.Contig10	74.90	573.39	2.94	0	**WRKY22**	CL4876.Contig1	5.74	1.14	-2.33	2.06E-18
	Unigene13091	1.26	5.44	2.11	1.13E-09		CL13979.Contig1	7.54	2.37	-1.67	1.22E-19
	CL898.Contig5	40.59	166.29	2.03	0		CL13979.Contig2	9.35	4.09	-1.19	2.22E-04
	CL898.Contig6	64.46	222.26	1.79	0		Unigene15232	7.16	3.20	-1.16	7.16E-16
	CL898.Contig3	36.85	121.77	1.72	1.02E-238	**WRKY4**	CL9110.Contig3	14.89	5.92	-1.33	1.13E-32
	CL898.Contig8	38.92	125.68	1.69	0	**WRKY46**	CL6991.Contig2	2.52	7.10	1.49	9.25E-13
	Unigene3765	2.53	6.52	1.36	5.29E-07	**WRKY70**	CL10835.Contig2	4.72	0.55	-3.10	1.97E-15
	CL898.Contig9	22.51	112.51	2.32	1.71E-116		Unigene12673	15.69	7.30	-1.10	2.33E-32

The prefix “CL” represents clusters, and “unigene” represents singletons. FPKM indicates the FPKM values of the unigenes in FS or OS. “Fold change” is equal to log_2_ (OS-FPKM / FS-FPKM). “+” indicates upregulated transcription, and “-” represents downregulated transcription.

## Discussion

### Sequence assembly

Since the Trinity method was developed [17], NGS has become widely used for *de novo* transcriptome analysis [14], especially in non-model plants lacking genome sequence information [17], such as *Reaumuria trigyna* [44], *Camelina sativa* [45] and *Hevea brasiliensis* [46]. In this study, we used Illumina RNA-Seq technology sequenced two libraries prepared from the FS and OS samples and obtained high quality of *de novo* assembly data. Further analysis indicated that the unigenes of *S*. *grandis* that were annotated and the expression patterns of *S*. *grandis* in response to grazing were calculated correctly.

This report is the first to comprehensively analyze the transcriptome and to identify the differentially expressed genes in *S*. *grandis* under grazing conditions without prior genome information. Prior to this study, Chen *et al*. (2009) used monocot rice plants, which have been entirely sequenced, as a model and simulated grazing by cutting and dabbing cow saliva to study the corresponding gene expression in response to grazing defoliation; however, that experiment only simulated grazing using rice but not wild plants in the actual habitat. Recently, this research group also reported defoliation treatment response in *Leymus chinensis* and *sorghum bicolor* plants [47–49]. In the present study, we selected *S*. *grandis*, a typical species found in the grasslands of Inner Mongolia [10], as our starting material, and using transcriptomic RNA-Seq analysis, we provided a complete gene expression profile for grazing, thus facilitating further studies on the complex responses of plants to grazing at the molecular level.

### The wound response in plants

Wounding is an inevitable threat for the survival of all organisms [50] that occurred more frequently under overgrazing condition. Many genes, such as those related to wounds and resistance to potential pathogen attacks, were activated in a grazing simulation study [51]. As a typical wound hormone [52], JA accumulates and modulates the expression of wound-associated genes in response to the plant’s wounds [53]. Reymond *et al*. (2000) analyzed the expression dynamics of 150 genes in mechanically wounded *Arabidopsis* leaves using a cDNA microarray technique and found that the genes involved in the synthesis or metabolism of members of the JA family are induced, including *LOX*, *FAD*, *AOS* and *OPR*. Chen *et al*. (2014) using the Illumina / Solexa platform sequenced seven cDNA libraries prepared from control, wounded (2, 6 and 24 h) and defoliated (2, 6 and 24 h) *L*. *chinensis* plants, by comparing the transcriptomic data, 1836 and 3238 genes were significantly differential expressed between wounding and defoliation treatment within one day. Among these genes, such as *LOX*, *AOS* and *OPR* were commonly activated in response of wounding and defoliation. Consistent with this observation, we also found that a number of the above genes, along with 2 *PLDs*, were upregulated by grazing (Table 3).

Wounding also induced *JAZ10* / *TIFY9*. In the core module (COI1–JAZs–MYC2) of the JA signaling pathway, the *JAZ* / *TIFY* genes are transcriptional targets of MYC2, and JA treatment quickly induces their transcripts, followed by the degradation of the SCF^COI1^-dependent proteasome and activation of the JA response [34, 35]. The activation of these genes indicates that the JA-dependent wounding response was likely activated. Repeatedly wounding the leaves of *Arabidopsis* resulted in stunted growth and increased endogenous JA content; however, these treatments did not stunt the growth of mutants that were deficient in JA synthesis, such as the *aos* and *opr3* mutants and the *fad3-2fad7-2fad8* triple mutant, indicating that wound-induced JA significantly suppresses plant growth [28]. Based on the literature and our own research, it was hypothesized that the wound response is enhanced by overgrazing, and wound-induced JAs are likely to participate in overgrazing-inhibited plant growth.

Additionally, *PLD* is involved in the response to drought stress [54], and JAs confer plants with the capacity to counter multiple biotic stimuli, such as pathogens [55]. These observations suggest that the complex interplay of gene expression patterns most likely also occurs under overgrazing conditions.

### The drought response in plants

In addition to damage the plant, grazing also reduces the soil’s water content [5]. Furthermore, water stress is an important participant in the plant’s response to mechanical wounding [50]; therefore, plants also suffer from drought stress under grazing conditions. In plants, CRT / DRE (C-repeat / dehydration-responsive element) is a cis-acting DNA regulatory element that initiates transcription in response to water deficiency [39]. In *Arabidopsis*, the expression levels of all of the CRT / DRE binding factors (CBFs / DREB1s) were low under normal growth conditions, but the transcripts were immediately enhanced following drought stress [56, 57]. *DREB1* overexpression in *Arabidopsis* not only strengthened the plant’s tolerance to drought but also resulted in plant growth retardation [58]. The constitutive expression of *Arabidopsis CBF1* in *Brassica napus* elevated drought tolerance [57]. The LEA (late-embryogenesis abundant) proteins, expressed by many prokaryotes and eukaryotes, are hydrophilic proteins associated with tolerance to dehydration [36, 37]. Many of the genes encoding the LEA proteins in *Arabidopsis* contain ABRE (ABA responsive element) and the DRE / CRT / LTRE element [37]. The expression of the LEA proteins are highly induced by water stress in peas (*Pisum sativum*) [36]. We also found that the transcripts of *CBF / DREB* and *LEA* were highly induced by overgrazing (Table 3), indicating that grazing involve in plant’s tolerance to dehydration.

The plant hormone ABA (abscisic acid) plays a critical role in the plant’s adaptation to abiotic environmental stressors (such as drought). During vegetative growth, ABA accumulates in the plant’s cells and regulates the expression of many genes during drought stress [59]. NCED (9-cis-epoxycarotenoid dioxygenase) is a key enzyme in ABA biosynthesis. *NCED* expression is induced, coinciding with an increased level of endogenous ABA in *Arabidopsis*, when exposed to drought stress [60]. *AtNCED3* overexpression in *Arabidopsis* confers improved drought tolerance via an increase in the ABA level, while *AtNCED3* antisense plants and the T-DNA insertion mutant show a drought-sensitive phenotype with lower ABA levels when compared to wild-type plants [60]. We observed that *NCED* and 6 *dehydrin* transcripts were increased by overgrazing (Table 3). Therefore, we hypothesize that the drought response in *S*. *grandis* may be activated and elevated drought tolerance when the plant is subjected to grazing.

### Plant immunity

Plants under grazing conditions are more likely to be attacked by pathogenic organisms when compared to non-grazing plants. On the one hand, the feces and urine deposition of livestock contain various microorganisms, and an increasing richness and abundance of microorganisms have been identified in soil following grazing due to trampling and fecal and urine deposition [61, 62]. On the other hand, the open wound damage to plant tissues caused by mechanical wounding provides a potential infection site for pathogen invasion [50, 52].

Plants use two innate immune system modes to protect against microbial pathogen attacks: pattern-triggered immunity (PTI), which is triggered by the perception of microbe-associated or pathogen-associated molecular patterns (MAMPs or PAMPs) through pattern recognition receptors (PRRs); or effector-triggered immunity (ETI), which is triggered by the recognition of pathogen effectors [63]. The perception of microbial pathogens by plants can be described in four phases using the “zigzag” model [64]. In the present study, six *FLS2* genes, which function as PRRs in plants to perceive bacterial flagella [41], showed upregulated expression following overgrazing (Table 4).

Many pathogen virulence effectors are secreted through the type III secretion system (TTSS) and are recognized by plants with corresponding *R* genes [55]. In *Arabidopsis*, the nucleotide-binding leucine-rich repeat (NB-LRR) classes of the R protein RPM1 confer resistance to *Pseudomonas syringae* strains expressing the avirulence genes *avrB* and *avrRpm1* [42, 65]; loss-of-function of *RPM1* plants show sensitive symptoms to *P*. *syringae* [66]. However, the RPM1-interacting protein RIN4, which is the target of the type III virulence effector and required for RPM1 activation [42], acts as a negative regulator of the plant’s basal defense response [67]. Consistent with this observation, we found that the transcripts of 11 *RPM1* genes were enhanced, whereas 4 *RIN4* genes were compromised by overgrazing (Table 4). We also observed that the genes required for *R* gene activities were induced by grazing. For example, SGT1, a component of SCF (Skp1-Cullin-F-box protein) ubiquitin ligases and a regulator, was active early in the plant’s *R* gene-mediated defense; the *SGT1b* mutation of *Arabidopsis* was defective in the plant’s early defenses [68]. Additionally, the molecular chaperone HSP90, which is critical for disease resistance in *Arabidopsis* due to its interaction with SGT1 [69], showed increased gene expression under the grazing condition. Therefore, we hypothesize that grazing elevates the resistance of *S*. *grandis* to pathogenic organisms.

Several transcription factors (TFs), including WRKYs, also show altered expression during ETI and PTI [70]. Accumulating data implicate the WRKY TFs in the plant immune response, acting as both positive and negative regulators of disease resistance [70]. For example, overexpression of the pathogen-inducible *OsWRKY31*, also known as *WRKY55* [71], enhances disease resistance in transgenic rice plants [72]. In contrast, WRKY7 and WRKY11 function as negative regulators in the plant defense response [73, 74]. In *Arabidopsis*, the loss-of-function *WRKY70* mutant showed enhanced disease susceptibility to *Erysiphe cichoracearum*, while *WRKY70-*overexpressing transgenic plants increased resistance to *E*. *cichoracearum* and suppressed the JA-induced defense response [75]. The altered expression of *WRKY* genes in present study (Table 4 and S3 Table) suggest that WRKY TFs may be an important component in the resistance of *S*. *grandis* to pathogenic organisms to provide grazing tolerance. However, the real roles of the WRKY TFs in *S*. *grandis* still need to be further identified. Based on the genes discussed above, we conclude that grazing not only subjects plants to biotic stressors and activates the plant’s immune response, it also confers resistance to pathogenic organisms.

In conclusion, to cope with the stressful occurrences that are associated with grazing, plants develop an avoidance / resistance strategy for optimal growth, such as size reduction. It is well documented that pathogens and abiotic stressors, such as wounding and drought stress, severely impact plant performance and productivity [28, 76, 77]. Dwarf plants under these stressors always show enhanced tolerance and altered gene expression [28, 58, 78]. Therefore, our observation supports the notion that the dwarf phenotypes of *S*. *grandis* are induced by overgrazing, which is at least partially caused by the additive effects of multiple stressors, including wounding, drought and immunity signals.

## Supporting Information

S1 Fig The growth status of *S*. *grandis* under the overgrazing and non-grazing conditions. (DOC)

S2 Fig The length distributions of the FS and OS contigs. (DOC)

S1 Table The primers used for the qPCR analysis. (XLS)

S2 Table The GO functional classification and the KEGG pathway analysis of the DEGs. (XLS)

S3 Table A summary of the genes related to abiotic stress and defense. (XLS)
