# Supplementary material for: De novo Assembly and Transcriptomic Profiling of the Grazing Response in Stipa grandis
Source: PLoS One. 2015 Apr 13;10(4):e0122641. doi: 10.1371/journal.pone.0122641 (PMC4395228; doi:10.1371/journal.pone.0122641)
Supplement: S1 Fig — (DOC) [file pone.0122641.s001.doc]

**Supporting information**


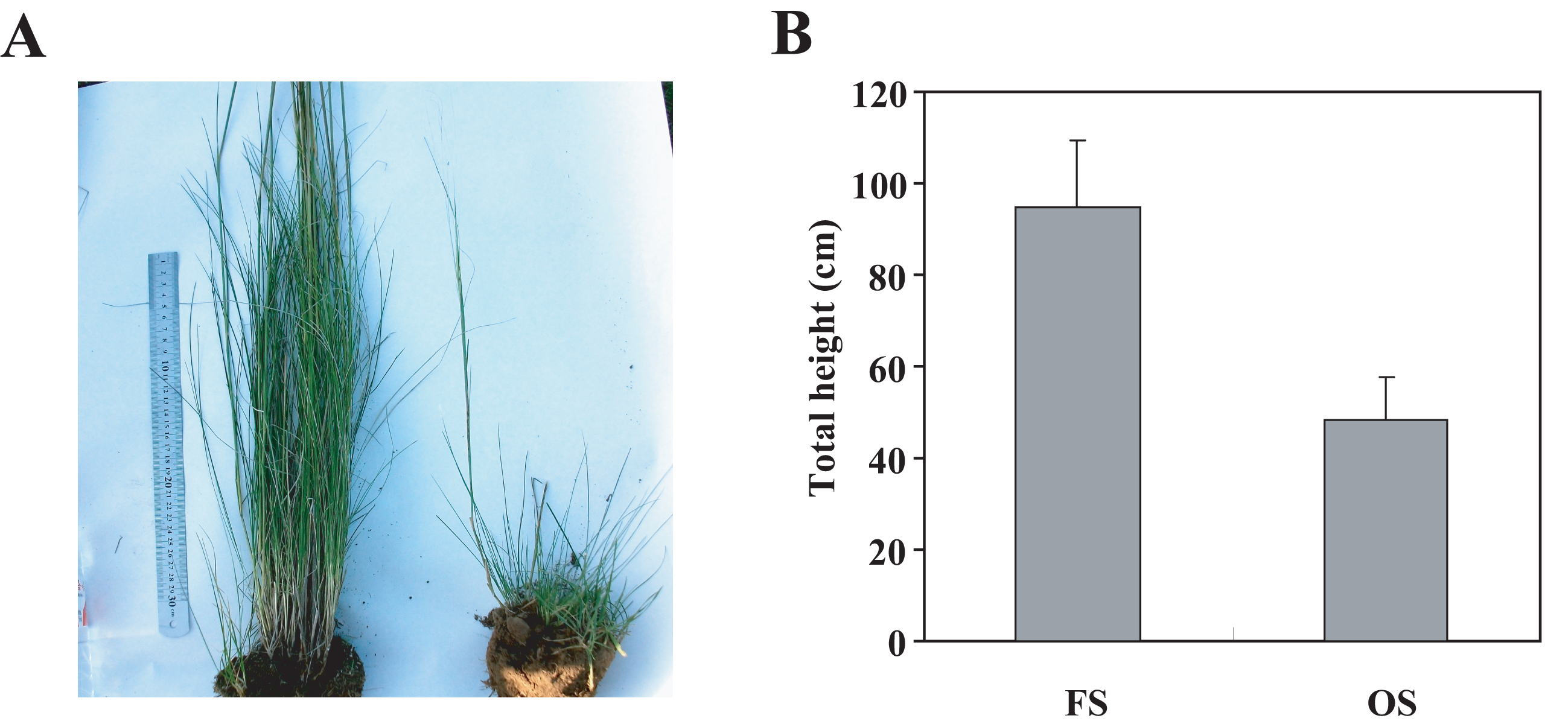


**S1 Fig. The growth status of *S. grandis* under the overgrazing and non-grazing conditions.** (A) Growth of the *S. grandis* under the overgrazing and non-grazing conditions. (B) Measurement of total height of *S. grandis* under the overgrazing and non-grazing conditions.
