# Supplementary material for: De novo Assembly and Transcriptomic Profiling of the Grazing Response in Stipa grandis
Source: PLoS One. 2015 Apr 13;10(4):e0122641. doi: 10.1371/journal.pone.0122641 (PMC4395228; doi:10.1371/journal.pone.0122641)
Supplement: S2 Fig — (DOC) [file pone.0122641.s002.doc]

**Supporting information**


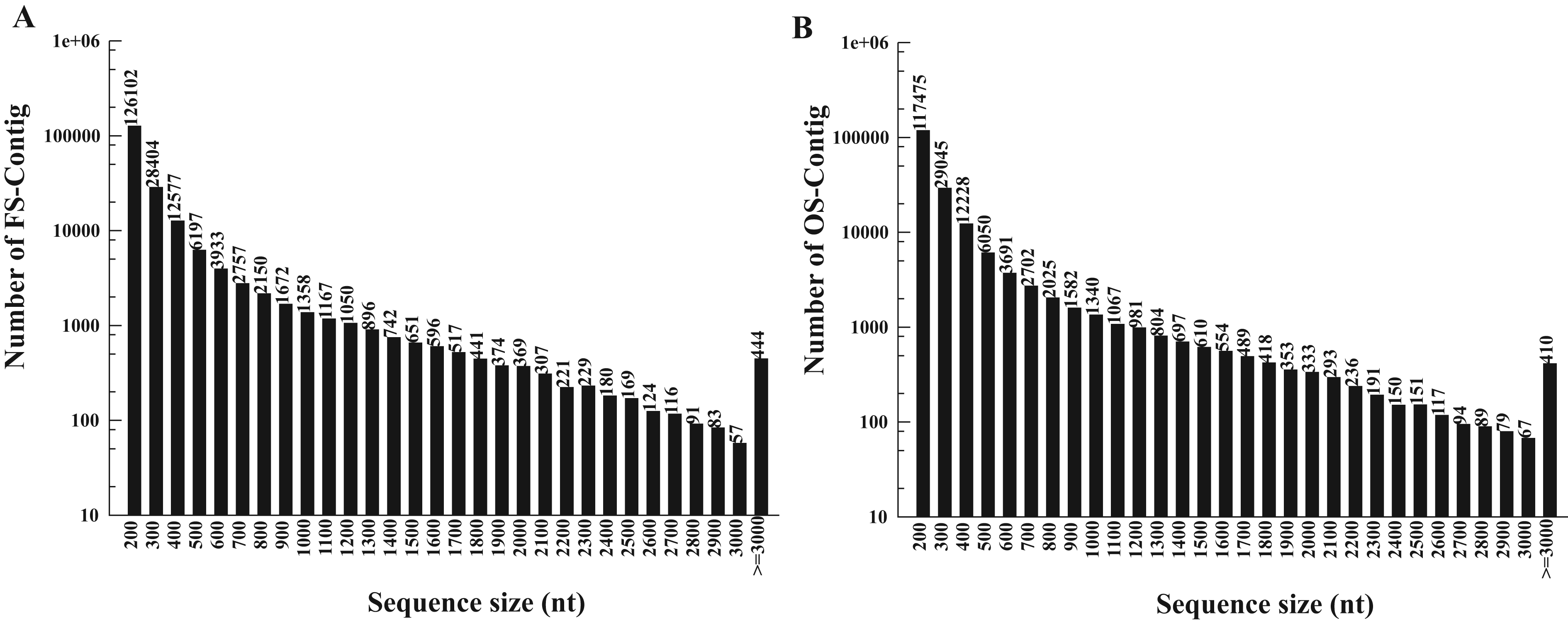


**S2 Fig. The length distributions of the FS and OS contigs.** Length distributions of (A) the FS contigs and (B) the OS contigs. The y-axis indicates the number of contigs, and the x-axis indicates the sequence sizes of the contigs.
